# Comparative Study between Traditional and Nano Calcium Phosphate Fertilizers on Growth and Production of Snap Bean (*Phaseolus vulgaris* L.) Plants

**DOI:** 10.3390/nano11112913

**Published:** 2021-10-30

**Authors:** Mona F. Abd El-Ghany, Mohamady I. El-Kherbawy, Youssef A. Abdel-Aal, Samaa I. El-Dek, Tarek Abd El-Baky

**Affiliations:** 1 Soil Department, Faculty of Agriculture, Cairo University, Giza 12613, Egypt; mohamady_mi@hotmail.com (M.I.E.-K.); youssefaabdelaal58@gmail.com (Y.A.A.-A.); altarek967@gmail.com (T.A.E.-B.); 2 Materials Science and Nanotechnology Department, Faculty of Postgraduate Studies for Advanced Sciences, Beni-Suef University, Beni-Suef 62511, Egypt; samaa@psas.bsu.edu.eg

**Keywords:** snap bean, monoammonium phosphate, nano calcium phosphate fertilizer, foliar application

## Abstract

Recently, nanofertilizers are being tested as a new technology, either for soil or foliar applications, to improve food production and with a reduced environmental impact. Nano calcium phosphate (NCaP) was successfully synthesized, characterized and applied in this study. A pot experiment was carried out in two successive seasons in 2016 and 2017 on (*Phaseolus vulgaris* L.) plants to obtain the best phosphorus treatments. The results were applied in a field experiment during the 2018–2019 season. Single superphosphate (SSP) at 30 and 60 kg P_2_O_5_ fed^−1^ and NCaP at 10%, 20% and 30% from the recommended dose were applied to the soil. Foliar application involved both monoammonium phosphate (MAP) at one rate of 2.5 g L^−1^ and NCaP at 5% and 10% from the MAP rate. The results of all experiments showed that NCaP significantly increased the shoot and root dry weights, the nutrient content in the shoot and root, the yield components, the nutrient concentration and crude protein percentage in pods of the snap bean plants compared with traditional P. The greatest increase was obtained from a 20% NCaP soil application in combination with a 5% NCaP foliar application. The present study recommends using NCaP as an alternative source of P to mitigate the negative effects of traditional sources.

## 1. Introduction

Agricultural land loses fertility as a result of human activity, which affects crop production and leads to starvation. One of the critical determinants of soil fertility is soil phosphorus availability [1]. Globally, more than 40% of soils suffer from low available phosphorus [2], and according to some estimates, there will be no phosphorus reserve in the soil by the year 2050 [3]. Plants absorb 15–20% of phosphate in fertilizer added to the soil, while 80–90% rapidly converts to low-available forms [4]. The soil of Egypt suffers from phosphorus deficiency [5] and applied phosphorus is retained in the soil due to many factors (i.e., clay minerals and high soil pH) [6]. Due to multiple problems associated with traditional phosphate fertilizers, nanofertilizers could be a suitable alternative. Nanofertilization relies on reducing bulk material to less than 100 nm to give a high surface area-to-volume ratio [7]. Nanofertilizers are more soluble and reactive relative to their traditional counterparts [8], easy to disperse with high tolerance for soil fixation [9], easily absorbed by plants, and released slowly to provide nutrients over long periods [10]. Liu and Lal [11] found nanohydroxyapatite to be such an alternative phosphate fertilizer, thereby showing that nanofertilizers help minimize the quantity of added fertilizer while reducing fertilizer loss and pollution due to agricultural malpractice [12,13,14,15].

In Egypt, the main sources of phosphorus are superphosphates and rock phosphates, but in recent years, water-soluble phosphorus fertilizers, such as monoammonium phosphate (MAP) and monopotassium phosphate (MPK), have been used along with traditional sources, especially during production of short-life vegetables such as the snap bean. These sources have high solubility and are popular in several countries for their high phosphorus content and excellent physical properties [16]. Essential plant nutrients are generally applied as a soil or foliar application to obtain maximum economic yields. For nutrients required in high quantities, soil application is more common and effective. However, under specific circumstances, foliar fertilization is more effective and economic; in intensified cultivation, it has become a necessary agrotechnical procedure.

Rane et al. [17] reported that calcium phosphate nanoparticles supplemented calcium and phosphate, the essential macronutrients required for profuse root proliferation. Calcium phosphate nanoparticles may help in the formulation of new nano growth promoter and nanofertilizers for agricultural use. Therefore, it could potentially help in reduction of the quantity of fertilizer applied to crops and contributing to precision farming, as it reduces fertilizer wastage and in turn environmental pollution due to agricultural malpractices.

Phosphorus is a critical and essential nutrient that limits the growth and production of plants, especially legumes, by affecting nodule formation and, thus, N-fixation [18]. In sustainable agriculture, the importance of legumes is increasing because they enhance the physicochemical properties of soil, increase organic matter content by leaving major quantities of crop residue and decrease the amount of nitrogen fertilizer. Snap bean (*Phaseolus vulgaris* L.) is one of the most important leguminous vegetable crops in the world. In Egypt, it is important for local consumption and second only to potatoes in the export trade [19]. It is a conventional food in the human diet, rich in protein, complex carbohydrates and vitamins, low in fat, and considered an important source of potassium, thiamine, selenium, molybdenum, folic acid and vitamin B6 [20]. The snap bean is one of the more sensitive crops to different kinds of environmental stressors, so it was selected for this study [21]. This study used the calcium phosphate nanoparticles that supplemented calcium and phosphate, the essential macronutrients required for profuse root proliferation (especially the snap bean, which absorbs a large quantity of P). Calcium phosphate nanoparticles may help in the formulation of new nano growth promoters and nanofertilizers for agricultural use. Therefore, it could potentially help in reduction of the quantity of fertilizer applied to crops and contributing to precision farming, as it reduces fertilizer wastage and in turn environmental pollution due to agricultural malpractices.

The goals of this paper are to compare the effects of soil and foliar applications of nano calcium phosphate (NCaP) with those of conventional phosphorus fertilizers single superphosphate (SSP) for soil application and monoammonium phosphate (MAP) for foliar application on growth, yield and nutrient contents of snap bean plants, and to evaluate the suitability of using nano calcium phosphate as a partial alternative to conventional phosphorous fertilizers.

## 2. Materials and Methods

To achieve the above goals, pot and field trials were conducted in private farm at El Agamian West El Fayoum Governorate, Egypt (29°20′12.7” N; 30°43′47.9” E; mean latitude 15 m above sea level). Daily temperatures range from 33.2 to 18 °C with an average of 25.7 °C, and daily relative humidity averages 73%, in a range between 79 and 68%. A pot experiment was carried out in two successive seasons: season one (SI, 2016–2017) and season two (SII, 2017–2018). An open greenhouse was used to evaluate the effect of different P fertilizer sources (traditional and nanophosphorus fertilizers) on snap bean plants and to obtain the best treatments to apply in a field experiment during the 2018–2019 season. Soil samples were taken from the site and analyzed for physical and chemical properties (Table 1) before starting the experiments, according to the methods described by Chapman and Pratt [22].

Uniform seeds of snap bean (*Phaseolus vulgaris.*) cv. Paulista were obtained from the Horticulture Research Institute, Agricultural Research Center, Ministry of Agriculture, Giza, Egypt. 

### 2.1. Materials Used

Single superphosphate (SSP) (as a traditional soil phosphate fertilizer, 12.5% P_2_O_5_), monoammonium phosphate (MAP) (as a traditional foliar phosphate fertilizer, 12-61-0) and nano calcium phosphate (NCaP) 18% P_2_O_5_ were used as soil or foliar applications.

#### 2.1.1. Nano Calcium Phosphate Preparation

Nano calcium phosphate particles were prepared at the centre of excellence for the production of nanomaterials, stdf 31305, using the co-precipitation method according to Mansour et al. [23]. Chemicals were used without purification; 0.3 M each of disodium hydrogen phosphate (Na_2_HPO_4_, Merck, Darmstadt, Germany) and calcium chloride (CaCl_2_·2H_2_O, Merck) (1:1) were used as starting materials. A diluted solution of ammonium hydroxide (NH_4_OH, Merck) or HCl (droplets) was used to adjust the pH to 5. The solutions were continuously stirred for 24 h. The pH value of the suspension was continuously adjusted and maintained at the above-mentioned values during precipitation. The solutions were then placed in Teflon flasks, tightly sealed and aged at room temperature for 24 h to allow precipitation. The white precipitate powder was filtered thoroughly and washed several times with double distilled water and finally dried at 50–60 °C for 12 h. The following equation represents the reactions:

Na_2_HPO_4_ + CaCl_2_·2H_2_O → CaHPO_4_·2H_2_O + 2NaCl

#### 2.1.2. Nano Calcium Phosphate (NCaP) Characterization

The prepared nano calcium phosphate particles were characterized using X-ray diffraction (XRD), using analytical-x0 pertpro (Almelo, the Netherlands) with Cu k α 1 target, λ = 1.5404 A°, 2θ ranging from 10 to 80 o, step size 0.02 o at 45 kV, 40 mA, to identify the phase composition and crystallinity of the calcium phosphate compounds. The FT–IR Spectrometer (FTIR RAMAN MODEL IS Bruker-Vertex RAM ll, Karlsruhe, Germany) was used to explore the chemical bonds and functional groups existing in the range of 4000–400 cm^−1^. A Field Emission Scanning Electron Microscope (Zeiss Sigma 500 VP Analytical FE-SEM Carl Zeiss, Oberkochen, Germany) was used to examine the prepared NCaP. The particle size and shape were studied using a transmission electron microscope (TEM) (JEOL-JME 2100, Tokyo, Japan).

### 2.2. Experimental Procedure

#### 2.2.1. Pot Experiment

Plastic pots of 30 cm inner diameter and 32 cm depth were filled with 10 kg soil. In each pot, 10 homogenous seeds were sown, and after full emergence, thinning was conducted to maintain four uniform seedlings per pot. The treatments were soil and foliar application with different sources of phosphorus fertilizer. Two sources were applied soil application, one using SSP as a traditional fertilizer at two rates (30 kg P_2_O_5_ fed^−1^ (recommended dose) and 60 kg P_2_O_5_ fed^−1^) and the other using NCaP at three rates (10%, 20% and 30% from the recommended dose). Foliar application used both monoammonium phosphate (MAP) as a traditional foliar application at the conventional dose of 2.5 g L^−1^ and with NCaP added at 5% and 10% from the conventional dose of MAP (2.5 g L^−1^). The study included 17 treatments (T1–T17) including the control treatment (T1) with three replications for each treatment in two seasons, SI (2016–2017) and SII (2017–2018). The details are shown in Table 2.

All phosphorus soil application treatments (SSP and NCaP) were applied after sowing, while foliar phosphorus fertilization treatments (MAP and NCaP) were sprayed at 20 and 30 days after sowing (DAS). A handheld manual sprayer (model 0417.02.00; Guarany Ind. & Com. Ltd. Lisboa, Portugal) was used to spray the different solutions of MAP and NCaP (approximately 100 mL per pot), and a few drops of Tween-20 were added to the spray solutions as a surfactant. For all plants that did not receive MAP, the amount of nitrogen found in MAP was calculated and sprayed to offset the effect of N in all treatments.

In addition, all pots received the recommended dose of nitrogen (40 kg N fed^−1^) from ammonium nitrate 33% N, before planting and 20 kg K_2_O fed^−1^ as potassium sulphate (50% K_2_O) of which 60% of the dose was added at planting and the remaining 40% 15 days after the first dose application.

#### 2.2.2. Field Experiment

Each plot area was 10.5 m^2^ (3 m × 3.5 m) and contained five ridges. Bean seeds (20 plants per m^2^) were planted on one side of the ridge in hills spaced 10 cm apart, and two seeds were sown in each hill. The plants were thinned to one plant per hill after 15 days. Soil preparation, fertilizer application (the recommended doses of N and K) and all other agricultural practices for snap bean production were followed according to the recommendation of the Egyptian Ministry of Agriculture. The experiment design was a randomized complete block (RCBD) with three replicates. According to the results of the pot experiment during the two seasons, the more efficient phosphorus fertilizer treatments were used in the field experiment. These included four treatments in addition to the traditional soil applications of 30 and 60 kg P_2_O_5_ fed^−1^ that were used for comparison.

All phosphorus soil application treatments (SSP and NCaP) were applied during soil preparation. Foliar fertilization (MAP and NCaP) spraying was done twice, at 30 and 45 days after sowing at a spray rate of 180 L fed^−1^. For all plants that did not receive MAP, the amount of nitrogen found in MAP was calculated and sprayed to offset the effect of N in all treatments.

### 2.3. Data Recording

In pot experiments, plants were harvested from each pot for biomass measurement and tissue analysis at 45 days after sowing. Shoots were cut just above the soil and roots were detached from the soil by soaking the root system in water and softly washing away the particles. The roots were sun-dried and then the shoot and root were oven dried at 70 °C, weighed to determine shoot and root biomass (g pot^−1^), and digested for subsequent tissue analysis. Nutrient concentrations (N, P and K) were determined in shoot and root as described by A.O.A.C [24]. The total nitrogen was determined by using the micro-Kjeldahl, phosphorus was determined spectophotomertricaly and potassium was determined using flame photometer.

For field experiment, at 70 days after sowing (harvest time) a random sample of 10 plants was selected for investigation in each plot to determine the mean values of the pod yield and its related parameters: the number of pods per plant, pod length (cm), pod diameter (mm), weight of pods per plant (g), weight of pod (g) and pod yield (ton ha^−1^). A total of 30 plants were fixed for each treatment, and the pods from each were collected and weighed. Nutrient concentrations (N, P and K) were determined in shoot and root as described by A.O.A.C [24]. The total nitrogen was determined by using the micro-Kjeldahl then, the nitrogen percentage was multiplied by 6.25 to estimate the crude protein percentages, phosphorus was determined spectophotomertricaly and potassium was determined using a flame photometer. 

### 2.4. Statistical Analyses

A randomized complete block design with three replications for each treatment was used according to Snedecor and Cochron [25]. The Least Significant Difference test (LSD) at a 5% level of probability was used to test the significance differences among the means. The MSTAT-C software package was used to carry out the statistical analysis [26].

## 3. Results and Discussion

### 3.1. Characterization of Nano Calcium Phosphate (NCaP)

#### 3.1.1. X-ray Diffraction Pattern

**Figure 1:**
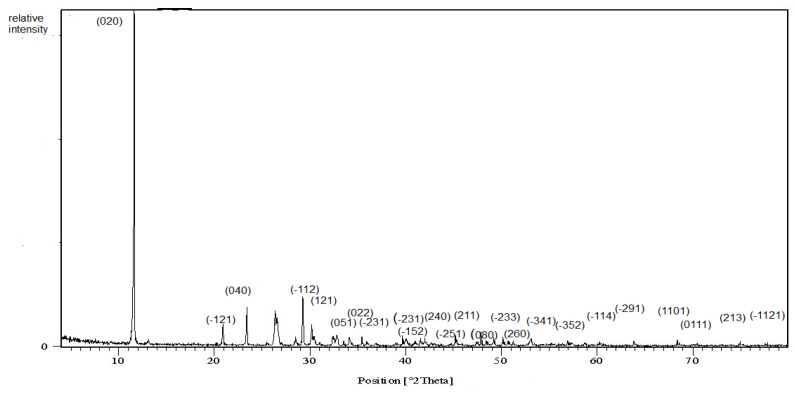
X-ray diffraction chart of the brushite.

The X-ray diffraction pattern of the prepared nanoparticles is illustrated in Figure 1. Clear reflections appeared and indexed on the chart. The d-spacing, 2 theta, relative intensities and Miller indices are reported in Table 3. The sample data were compared easily and indexed using the ICDD card number 00-011-0293 of calcium phosphate hydroxide hydrate. The crystalline nature of the nanoparticles was cleared as identical to the brushite mineral of monoclinic symmetry with four molecules per unit cell. The broadening of the diffraction peak assured the fine particle size. The crystallite size was calculated using the well-known Scherrer’s Equation [27]:

(1) $$T=\frac{K\;\lambda }{\beta \cos\theta }$$

where T is the crystallite size as calculated from the (020) plane; Κ is the dimensionless shape factor (0.9), which varies with the actual shape; λ is the target X-ray wavelength; β is the line broadening in radians at half the maximum intensity after subtracting the instrumental line broadening; and θ is the Bragg angle. In our case, the crystallite size was calculated to be 15.7 nm 

#### 3.1.2. Scanning Electron Microscopy (SEM)

**Figure 2:**
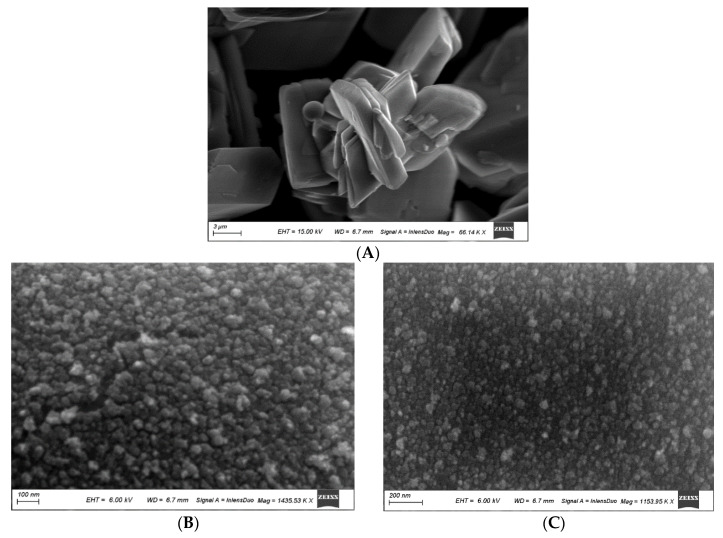
Scanning Electron Micrograph (SEM) of brushite phase (**A**), Scanning Electron Micrograph (SEM) of brushite phase at two different magnifications (**B**,**C**).

Scanning electron micrographs (SEM) are presented in Figure 2A. Clear, large geometric sheets of monoclinic structures are seen on small scales that define the grain shape. Scanning electron micrographs (SEM) are presented in Figure 2B,C. More focusing of the surface morphology reveals ultrafine grains with sharp boundaries ranging from 10 to 16 nm. The agglomeration is clear, and very small pores are rare. The grains are well defined regarding their shape, size and distribution.

#### 3.1.3. Transmission Electron Microscopy (TEM)

**Figure 3:**
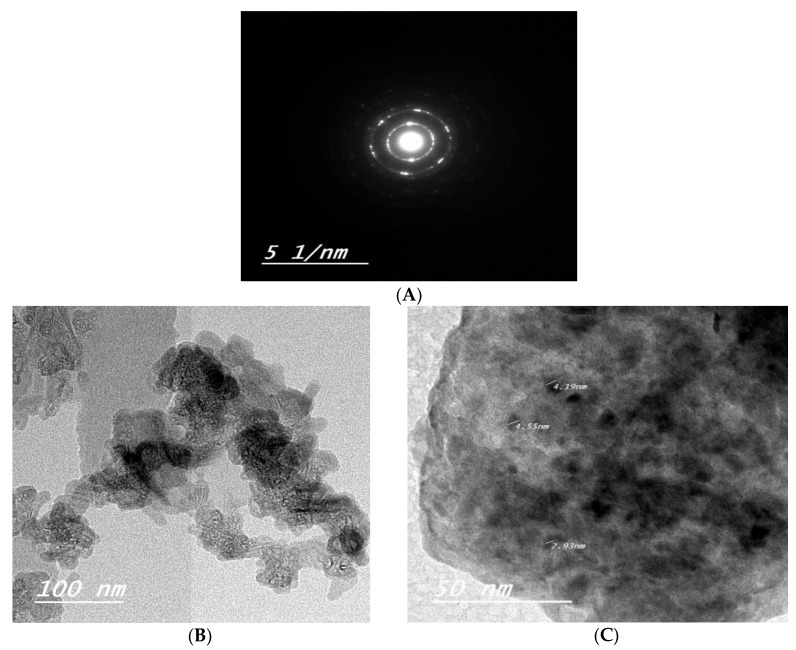
Selected area electron diffraction of Calcium Phosphate Nanoparticles (**A**),High Resolution Transmission electron micrographs at two different magnifications of Calcium Phosphate Nanoparticles (**B**,**C**).

The selected area electron diffraction (SAED) shown in Figure 3A assured excellent crystallinity, despite the small crystallite size where clear concentric rings were observed easily. Moreover, the preferred orientation agrees well with the monoclinic symmetry in XRD (Figure 1). High-resolution transmission micrographs (TEM) are presented in Figure 3B,C. The average particle size measured on the micrograph was about 10 nm. The particles appear agglomerated due to the absence of a surfactant and/or capping agent during preparation.

#### 3.1.4. Fourier Transformer Infra-Red (FTIR)

**Figure 4:**
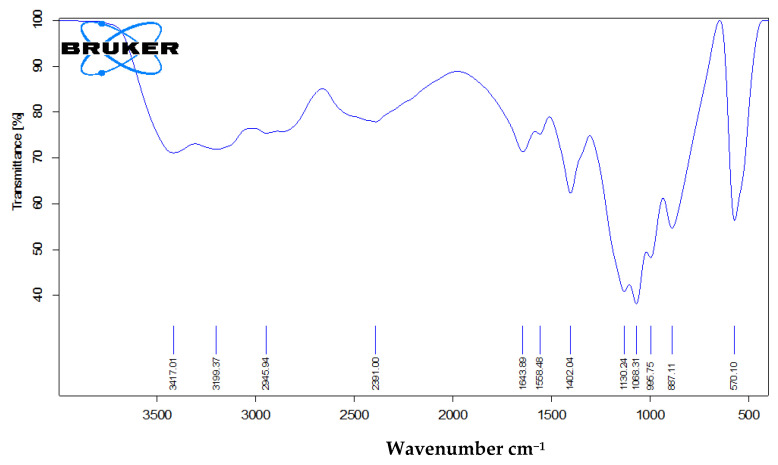
Fourier Transformer Infrared (FT–IR) spectrum of nanocalcium phosphate.

An examination of calcium phosphate nanoparticles using FTIR showed that the surface of the prepared nanoparticles carries several types of functional groups, including P–O–P bonding, two types of phosphate anions (HPO_4_^2−^ and PO_4_^3−^), –OH and H_2_O. More details of the surface functional groups are shown in Figure 4 and Table 4.

### 3.2. Pot Experiment

#### 3.2.1. Effect of Traditional and Nano Calcium Phosphate Fertilizers on Shoot and Root Dry Weights of Snap Bean Plants

**Figure 5:**
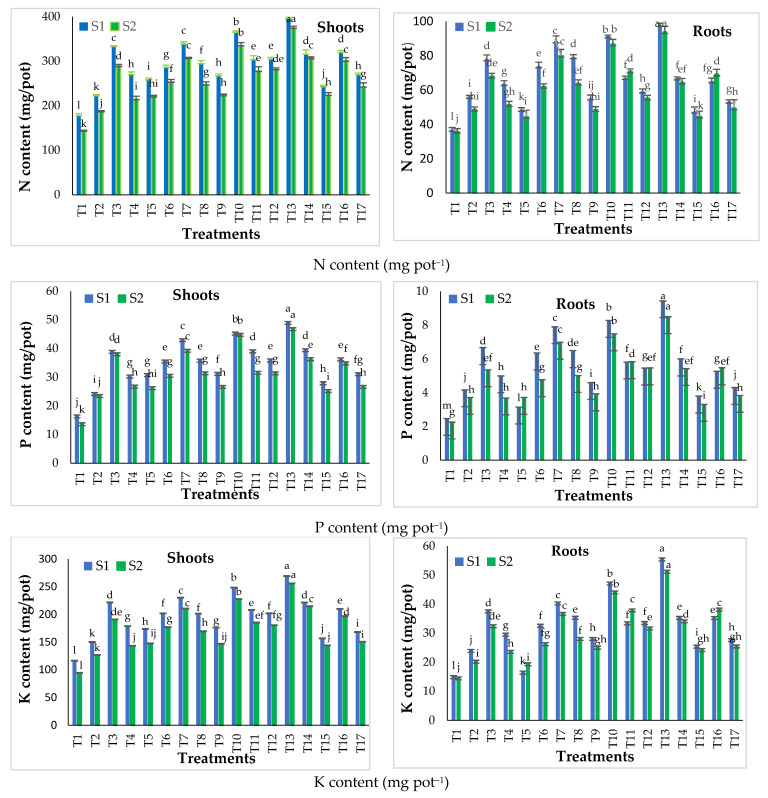
Effect of different phosphorus treatments on the nutrients content in shoot and root of snap bean plants at 45 days after sowing during the two seasons: S1 = season 2016–2017 and S2 = season 2017–2018. Data presented are means ± SD (*n* = 3). Different letters mean values indicate significant differences at *p* ≤ 0.05, according to the least significance difference.

A comparison between traditional (SSP and MAP) and nano calcium phosphate (NCaP) fertilizers on the growth and nutrient status of snap bean plants (*Phaseolus vulgaris* L.) was conducted through a pot experiment in two seasons (2016–2017 and 2017–2018) to determine the optimal source and concentrations of the phosphorus sources tested. For this purpose, shoot and root dry weights (Table 5) and nutrient status (Figure 5) were assessed. The results presented in Table 5 generally show that, in both seasons, the shoot and root dry weights (g pot^−1^) increased (*p* ≤ 0.05) for all phosphorus treatments compared to those of the control plants (T1, soil application of SSP at the recommended rate of 30 kg P_2_O_5_ fed^−1^). The highest values for both seasons were recorded in the following order: T13 > T10 > T7 > T3. The lowest values were obtained from the traditional phosphorus fertilizer T1 (control) in both seasons. The shoot and root dry weights in both seasons recorded by T13 (soil application of 20% NCaP of the recommended rate with 5% NCaP as foliar application) increased (*p* ≤ 0.05) relative to all other phosphorus treatments. 

As shown in Table 5, the conventional fertilizer (SSP) was tested at different rates (T1–T8); the results showed that both shoot and root dry weights increased (*p* ≤ 0.05) with an increasing phosphorus fertilizer rate from 30 (T1–T4) to 60 (T5–T8) kg P_2_O_5_ fed^−1^ as soil application with or without foliar application in both seasons. These results agree with those of Turuko and Mohammed [31], who reported that phosphorus was important for cell division. Accordingly, increased plant height, number of branches and leaf area, which in turn increased the photosynthesis area and, consequently, the dry weight.

The data in Table 5 also revealed, irrespective of SSP rates, the addition of foliar application of traditional MAP and NCaP at 5% and 10% of the recommended MAP rate increased (*p* ≤ 0.05) shoot and root dry weights relative to soil application of SSP alone in both seasons. Foliar application of phosphorus with NCaP at both concentrations (5% and 10% from MAP) performed better than MAP in improving plant growth. The highest values of shoot and root dry weights were recorded with foliar application at 5% NCaP followed by 10% NCaP, then MAP at both 30 and 60 kg P_2_O_5_ fed^−1^ in both seasons (Table 5). These results agreed well with Rady et al. [32], who reported that foliar application of *Phaseolus vulgaris* L. plants with NCaP was better than with MAP. Amira Sh. Soliman et al. [33] reported that growth parameters of baobab (*Adansonia digitata* L.) were (*p* ≤ 0.05) enhanced by hydroxyapatite nanoparticles followed by diammonium phosphate (DAP) and monoammonium phosphate (MAP) compared to control plants. They recommended applying hydroxyapatite nanoparticles in foliar application to increase growth and nutrition. 

The results also indicated that the beneficial effects of foliar application (MAP, 5% and 10% NCaP) in both seasons were more pronounced for shoot and root dry weights in plants treated with 30 rather than 60 kg P_2_O_5_ fed^−1^ (Table 5). In the first season, the shoot increased by 23.38%, 73.76% and 42.59% and the root by 43.62%, 94.63% and 62.42% after treatment with 30 kg P_2_O_5_ fed^−1^ in combination with foliar application by MAP, 5% NCaP and 10% NCaP, respectively, compared to plants treated only with 30 kg P_2_O_5_ fed^−1^. For plants treated with 60 kg P_2_O_5_ fed^−1^ in combination with foliar application by MAP, 5% NCaP and 10% NCaP, the values of increase for the shoot were 7.39%, 24.00%, and 10.75% and for the root, 31.53%, 52.71%, and 38.91%, respectively, over the plants treated only with 60 kg P_2_O_5_ fed^−1^.

The corresponding values for the second season were 28.57%, 85.25%, and 40.28% in the shoot and 31.91%, 80.85%, and 37.59% in the root of plants treated with 30 kg P_2_O_5_ fed^−1^ in combination with foliar application by MAP, 5% NCaP and 10% NCaP, respectively, over the plants treated only with 30 kg P_2_O_5_ fed^−1^ as soil application. For plants treated with 60 kg P_2_O_5_ fed^−1^, the percentage increase reached 10.87%, 32.28% and 9.13% in the shoot and 28.40%, 65.09% and 34.91% in the root in combination with foliar application by MAP, 5% and 10% NCaP, respectively, over the plants only treated with 60 kg P_2_O_5_ fed^−1^ (Table 5). 

The data depicted in Table 5 show that the shoot and root dry weights increased (*p* ≤ 0.05) relative to the conventional fertilizer treatment (T1) in both seasons because of the application of NCaP fertilizers (T9–T17). These results agreed with those of Amira Sh. Soliman et al. [33] and Abdel-Salam [34]. This growth improvement may be due to the beneficial effects of nanoparticles, which have high reactivity resulting from a greater density of reactive areas and more specific surface area [33]. In addition, hydroxyapatite nanoparticles activated the growth of the root zone, which allowed the plant to take up water and nutrients more efficiently [35], leading to increases in chlorophyll, carbohydrates and carotenoids [33], which in turn improve plant growth.

The results also showed that NCaP, at a rate of 20% soil application, achieved the highest values of shoot and root dry weights without or with foliar application of NCaP fertilizer at 5% and 10% (T12, T13 and T14, respectively) followed by NCaP at the rate of 10% soil application without or with foliar application of NCaP fertilizer at 5% and 10% (T9, T10 and T11, respectively). However, NCaP at the rate of 30% soil application without or with foliar application of NCaP fertilizer at 5% and 10% (T15, T16 and T17, respectively) recorded the lowest values in both growing seasons, but was still higher than that of the control plants. Inhibition of growth was observed in the snap bean plants treated with NCaP at the highest concentration (30% of recommended P dose) compared to the other NCaP rates (Table 5). The results agreed with those of Singh et al. [14], who reported that nanoparticles permeate into the seed easily and raise available nutrients to the seedling, which increased shoot and root growth; however, if the concentration were more than the optimum, germination and growth of the seedling was inhibited. Priya et al. [36] observed increased germination and growth of seedlings with phosphorus nanoparticles concentration, but up to certain limits. The reduction in shoot and root lengths at higher doses may be attributed to toxic levels of nanoparticles [36,37]. Turuko and Mohammed [31] observed that high dose of phosphorus fertilizer tended to form nutrient interactions that may have affected the availability of other nutrients essential for growth. Arshad et al. [38] reported that high concentration of TiO_2_ NPs could reduce the plant biomass, height and chlorophyll content, which might be due to the toxic impact of nanoparticles on plants, and damages leaf membrane and production of hydrogen peroxide, as shown by Waani et al. [39].

Foliar application of NCaP (5% and 10% from the recommended rate of MAP) in combination with NCaP soil application increased (*p* ≤ 0.05) shoot and root dry weights relative to soil NCaP application alone in both seasons irrespective of NCaP rate (Table 5). That may have been due to the effect of applying foliar nanofertilizers on the neuromas of vital processes inside the plant organs, which increased plant growth and increased nutrient absorption [40].

In both seasons of the experiment, the soil or foliar application of NCaP performed better than the conventional phosphate fertilizer SSP for soil application and MAP for foliar application at improving shoot and root dry weights (g pot^−1^). The effect of T5 was approximately equal to the effect of T9, and both were higher than that of SSP (T1), namely, 10% NCaP of the recommended rate, equivalent to 60 kg P_2_O_5_ fed^−1^ as SSP (traditional). Therefore, nanofertilizers could potentially help minimize the quantity of fertilizer added to crops [12,13,14,15].

#### 3.2.2. Effect of Traditional and Nano Calcium Phosphate Fertilizers on the Nutrient (N, P and K) Content of Snap Bean Plants

The results presented in Figure 5 clearly reveal that, in both seasons, all treatments increased (*p* ≤ 0.05) nitrogen, phosphorus and potassium content in the shoot and the root of snap bean plants compared to the control treatment (T1). The results are in good accordance with those reported by Youssef et al. [41], who found that different phosphorus sources affected N, P, K, Ca, Fe and Mn concentrations in the leaves.

In this regard, the results showed that increased nutrients content in the shoot andthe root of snap bean plants (Figure 5) were closely linked to an increase in vegetative growth (Table 5). These results are in harmony with those of Zahra et al. [42], who reported that the maximum increase in the shoots and roots lengths reached 15.9 ± 0.3% and 3.8 ± 0.3% respectively, which was concurrent with improved P content in the plants. In this respect, Youssef et al. [41] reported that the increase in N, P and K stimulated photosynthesis, which lead to improvements of the vegetative growth. The addition of phosphorus led to an increased root-to-shoot ratio and boosted the growth of lateral roots [43], which resulted in enhanced water and nutrient absorption. In addition, hydrolysis of the terminal phosphate group in adenosine triphosphate (ATP) during conversion to adenosine diphosphate (ADP) provided the energy for many plant processes, including water and nutrient absorption [44].

Concerning the conventional fertilizer treatments (T1–T8), data in Figure 5 show that N, P and K content in the shoot and root increased (*p* ≤ 0.05) with increasing phosphorus applied from 30 to 60 kg P_2_O_5_ fed^−1^ as SSP in both seasons. These results are in harmony with those of Ehiagiator et al. [45], who reported that a higher application of phosphorus may expose the plant nutrient to a larger surface area, thereby boosting nutrient absorption. The highest content of N, P and K in the shoot and root were recorded by conventional fertilizer treatments for T7 (60 kg P_2_O_5_ fed^−1^ as soil application with 5% NCaP as foliar application). Moreover, the results showed that the foliar application of MAP (as a traditional phosphorus foliar application), 5% and 10% from the recommended rate of MAP, as NCaP in combination with SSP soil application at both 30 and 60 kg P_2_O_5_ fed^−1^ increased (*p* ≤ 0.05) N, P and K contents in the shoot and root compared to soil application alone in both seasons. This may have resulted from the synergistic effects of both soil and foliar phosphorus applications. The highest values of N, P and K contents in the shoot and root of were recorded with foliar application at 5% NCaP followed by 10% NCAP, then MAP at both 30 and 60 kg P_2_O_5_ fed^−1^ in both seasons (Figure 5). These results agree with those of Rady et al. [32]

Regarding NCaP fertilizer treatments (T9–T17), the data in Figure 5 show that its application as a P source increased (*p* ≤ 0.05) N, P and K contents in the shoot and root compared to T1 (control) in both seasons. The results agreed well with Abdel-Salam [34] and El-Kereti et al. [46], who observed that greater density in reactive areas of nHA (nanohydroxyapatite) particles increased the uptake of phosphorus. This, in turn, enhanced the leaf moisture percentage, total chlorophyll, total carbohydrates, crude protein and nutrient concentration. Nanoparticles with a diameter less than the pore diameter (5–20 nm) of a plant cell wall easily entered to reach the plasma membrane [47]. NHA raised the usage efficiency of phosphorus by different mechanisms, such as slow or controlled release, targeted delivery and low molecule diameter.

The results presented in Figure 5 also show that the optimal concentration of NCaP soil application with or without foliar application was 20% from the traditionally recommended dose of 30 kg P_2_O_5_ fed^−1^ from SSP, and the concentration of 5% of NCaP foliar application was better than 10% of NCaP. Results showed that the best NCaP treatment was T13 (20% NCaP of the recommended rate as soil application with 5% NCaP as foliar application) in the shoot and the root of snap bean plants in both seasons. 

Finally, the results of the pot experiment in both seasons clearly showed that the highest values of shoot and root dry weights and N, P and K content were recorded by NCaP treatments. The highest values were obtained by T13 (20% NCaP of the recommended rate as soil application with 5% NCaP as foliar application), followed by T10 (10% NCAP of the recommended rate as soil application with 5% NCaP as foliar application). Conversely, for conventional fertilizer treatments, the highest values of shoot and root dry weights and N, P and K content in the shoot and root were obtained by T7 (60 kg P_2_O_5_ fed^−1^ as soil application with 5% NCaP as foliar application) followed by T3 (30 kg P_2_O_5_ fed^−1^ as soil application with 5% NCaP as foliar application). Therefore, T3, T7, T10 and T13 displayed the best results and were selected for the field experiment in addition to T1 (control) and T5 (traditional) for comparison purposes.

### 3.3. Field Experiment

#### 3.3.1. Effect of Traditional and Nano Calcium Phosphate Fertilizers on the Yield and Components of Snap Bean Plants

The results presented in Table 6 reveal that all P treatments (*p* ≤ 0.05) enhanced the yield and its components of snap bean plants compared to the traditional soil application (SSP; 30 kg P_2_O_5_ fed^−1^) in the following descending order: T13 > T10 = T7 > T3 > T5 > T1. Increasing P rates from 30 to 60 kg P_2_O_5_ fed^−1^ significantly increased (*p* ≤ 0.05) the number of pods per plant, pod length (cm), pod diameter (mm), weight of pods per plant (g), weight of pod (g) and pod yield (ton ha^−1^). This result agreed with El-Azizy et al. [41], who found that the enhanced yield after an application of phosphorus fertilizer indicated a strong relationship between dry matter accumulation and plant yield [29]. This may have been because the added phosphorus increased the number of branches per plant as well as the leaf area, which increased the photosynthesis area and the number of pods per plant [29].

The data in Table 6 also showed that the combined treatments T3 and T7 (combination of soil application SSP at both 30 and 60 kg P_2_O_5_ fed^−1^, respectively, with 5% NCaP as foliar application) were more effective than individual soil treatments T1 and T5 (30 and 60 kg P_2_O_5_ fed^−1^, respectively) at improving the yield and components of snap bean plants.

The results clearly showed that yield components were affected by different phosphorus sources. Significant increases (*p* ≤ 0.05) in the number of pods plant^−1^, pod length (cm), pod diameter (mm), pod weight (g plant^−1^), weight of pod (g) and pod yield (ton ha^−1^) due to the NCaP fertilizer compared with traditional P sources (SSP). The maximum values for all studied yield were obtained with T13 (20% of traditional soil application as SSP with 5% of traditional foliar application as MAP through NCaP fertilizer), which increased (*p* ≤ 0.05) relative to all other phosphorus treatments (Table 6). At either the low or high rate, NCaP was more effective than traditional P at increasing plant yield and its components. The results were in agreement with those reported by Abdel-Salam [34], who reported that a high rate of NCaP gave higher numbers of pods and a higher seed yield for faba bean plants compared to a high rate of triple super phosphate. Liu and Lal [11] stated that NCaP fertilizer enhanced the growth and seed yield of soybeans compared with traditional P fertilizer. Taskin et al. [48] reported that, compared to a soluble source of phosphorus, NCaP increased the growth and P uptake in lettuce plants grown in calcareous soil [49].

The values for the number of pods plant^−1^, pod diameter (mm), pod weight (g plant^−1^), weight of pod (g) and pod yield (ton ha^−1^) for T10 (10% NCaP soil application with 5% NCaP foliar application) were approximately equal to the values for T7 (60 kg P_2_O_5_ fed^−1^ soil application as SSP with 5% NCaP foliar application). That meant that 10% NCaP of the recommended rate was equivalent to 60 kg P_2_O_5_ fed^−1^ as SSP (traditional) both applied as soil application. As previously mentioned, nanofertilizer has an edge over conventional fertilizer due to the controlled release of nutrients and the lower amount of fertilizer, which increases nutrient use efficiency (NUE) and reduces production costs [9]. 

#### 3.3.2. Effect of Traditional and Nano Calcium Phosphate Fertilizers on the Nutrient Concentrations and Crude Protein Percentage in Pods of Snap Bean Plants

The data in Table 7 shows that nitrogen, phosphorus and potassium concentrations and percent of crude protein in snap bean pods increased with increasing P rates from 30 to 60 kg P_2_O_5_ fed^−1^. This result agreed with El-Azizy et al. [40], who found that increasing applications of phosphorus increased significantly the nutrient concentration and protein content of broad bean seeds. Foliar application of 5% NCaP in combination with soil application of SSP increased significantly (*p* ≤ 0.05) N, P, K and crude protein percentage compared with applying SSP alone at both rates. Kaviani et al. [50] reported that foliar application of NCaP had a significant positive effect on the leaf and N, P and K content compared with traditional P fertilizer.

The results of the nutrient concentration and crude protein percentage in snap bean pods were increased (*p* ≤ 0.05) by all NCaP fertilizer treatments compared to traditional treatments T1 and T5. The results agreed with Burhan and Al-hassan [49]. Soil application of NCaP at both 10 and 20% with 5% foliar application (T13 and T10) achieved the highest pod concentration of P and K compared to SSP at both rates with and without foliar application (T1, T5, T3 and T7). This may have been due to the positive interaction between phosphorus and potassium as observed by Rajonee et al. [51]. NCaP fertilizer gave higher concentrations of N, P and K and crude protein percentage compared to a high rate of SSP (60 kg P_2_O_5_ fed^−1^).

These results agreed with those of Dhansil et al. [52], who found that the nutrient content of a pearl millet crop increased significantly when using both NP and traditional phosphorus fertilizers. The highest nutrient and crude protein contents were obtained from the application of a 2.5 times reduction in the recommended dose of phosphorus through NP fertilizer [52]. The slow and steady release of nutrients from nanofertilizer regulated the release of nutrients from the fertilizer and minimized losses resulting in the increased uptake of nutrients. Because phosphorus is critical for root growth, density and length [53,54], its further acquisition improves the symbiotic relation between rhizobium and legume roots [55]; hence, it causes increased nodulation, nitrogen fixation and, therefore, nitrogen content [56]. In this respect, Hagagg et al. [57] recommended nano NPK supplements to increase fertilizer efficiency. They wrote that nanofertilizers promote the uptake of water and nutrients, which is reflected in plant growth. Moreover, nanofertilizers have a huge surface compared to conventional fertilizers, and this increases the plant’s metabolic efficiency. The small diameter of the nanoparticles (25–50 nm) results in increased total surface area and decreased phosphorus fixation. The controlled release of nutrients and increased phosphorus uptake allow more phosphorus to be available for a longer time [52].

The use of nanofertilizer to control the release of nutrients can be considered an effective way to achieve sustainable agriculture and preserve the environment. Some soluble phosphate salts highly affect phosphorus fertilizers that are used extensively in agriculture, which leads to higher eutrophication of surface waters, while solid phosphates are less effective in supplying phosphorus. In contrast, NCaP fertilizers could supply sufficient phosphorus but with less mobility in the environment [11].

## 4. Conclusions

It can be concluded that both soil and foliar applications of NCaP fertilizers increased plant growth and yield components better than traditional phosphate fertilizers did. Furthermore, the combined effect of soil and foliar NCaP fertilizers recorded the highest values for vegetative growth, nutritional value and yield of snap bean plants. The optimal concentration of NCaP fertilizer as a soil application, with or without foliar application, was 20% from the traditional recommended dose (30 kg P_2_O_5_ fed^−1^ from SSP), while a concentration of 5% NCaP of an MAP dose was optimal for foliar application. It seems that using nanofertilizers not only reduces environmental pollution but also penetrates more deeply into the roots and leaves, thus improving the physiological traits and yield of snap bean plants. Therefore, the present study recommends using nanofertilizers as an alternative phosphorus fertilizer. However, a detailed physiological and molecular understanding of its impact on the snap bean plant is needed to validate its prospective application in agriculture. These studies demonstrate the need for further research with long-term field studies to examine NCaP regarding water and nutrient uptake, translocation and interactions with the other elements, particularly as influenced by soil properties.

## Figures and Tables

**Table 1:** Some physico-chemical properties of soil used for the three seasons.

Properties	Particle Size Distribution				pH 1:2.5	ECe dSm^−1^	Soluble Anions (meq^−1^)			Soluble Cations (meq^−1^)				Available Macronutrients (mg kg^−1^)		
	Sand (%)	Silt (%)	Clay (%)	Soil Texture			HCO_3_^−^	Cl^−^	SO_4_^−^	Na^+^	K^+^	Ca^2+^	Mg^2+^	N	P	K
	Pot experiment															
SI (2016/17)	77.40	15.25	7.35	Sandy loam	7.70	0.96	5.90	2.87	0.83	2.75	0.90	3.50	2.45	25.00	6.30	194.00
SII (2017/18)	76.80	14.75	8.45	Sandy loam	7.60	0.94	6.90	1.90	1.20	2.40	0.80	3.70	2.30	27.00	7.00	210.00
	Field experiment Season (2018–2019)															
	77.20	15.00	7.80	Sandy loam	7.80	0.95	6.50	2.20	0.80	2.51	0.75	3.54	2.70	23.00	5.30	187.00

**Table 2:** The treatments of traditional and NCaP fertilizers tested at SI (2016–2017) and SII (2017–2018) seasons.

* P-Fertilizer	Treatment	Rate
**SSP**	**T1**	30 kg P_2_O_5_ fed^−1^ (recommended dose) as soil application (control)
	**T2**	T1 +2.5 g/L MAP as foliar application
	**T3**	T1 + 5% NCaP as foliar application
	**T4**	T1 + 10% NCaP as foliar application
	**T5**	60 kg P_2_O_5_ fed^−1^ as soil application
	**T6**	T5 + 2.5 g/L MAP as foliar application
	**T7**	T5 + 5% NCaP as foliar application
	**T8**	T5 + 10% NCaP as foliar application
**NCaP**	**T9**	10% NCaP (of the recommended rate) as soil application
	**T10**	T9 + 5% NCaP as foliar application
	**T11**	T9 + 10% NCaP as foliar application
	**T12**	20% NCaP (of the recommended rate) as soil application
	**T13**	T12 + 5% NCaP as foliar application
	**T14**	T12 + 10% NCaP as foliar application
	**T15**	30% NCaP (of the recommended rate) as soil application
	**T16**	T15 + 5% NCaP as foliar application
	**T17**	T15 + 10% NCaP as foliar application

* SSP = single super phosphate; NCaP= nanocalcium phosphate; and MAP = monoammonium phosphate.

**Table 3:** Values of d-spacing, 2 theta, relative intensity and Miller indices (hkl) of the sample as extracted from the X-ray data analysis.

Pos. [°2Th.]	d-Spacing [Å]	Rel. Int. [%]	(hkl)
11.6415	7.60166	100	(020)
20.9303	4.24437	6.56	(−121)
23.3968	3.80222	11.11	(040)
29.2588	3.05242	14.30	(−112)
30.4671	2.93406	2.64	(121)
33.5449	2.67157	1.15	(051)
34.0854	2.63043	2.54	(022)
35.4079	2.53516	2.09	(−231)
39.7037	2.27021	2.19	(−161)
41.5395	2.17402	2.34	(−152)
42.0094	2.15079	2.43	(240)
42.8994	2.1082	0.50	(−251)
44.7010	2.02734	0.55	(211)
45.1943	2.00635	3.07	(−123)
47.8587	1.90069	4.14	(080)
49.232	1.85084	2.27	(−233)
50.2017	1.81583	2.46	(260)
50.6988	1.80068	1.53	(−262)
51.2680	1.78202	1.67	(−181)
53.0611	1.72595	2.00	(−341)
56.9298	1.61751	1.60	(−352)
58.7486	1.5717	0.82	(222)
60.3067	1.53477	0.76	(−114)
63.8810	1.45724	1.38	(−291)
68.4063	1.37146	1.75	(1101)
70.2666	1.33964	0.26	(0111)
74.9556	1.26704	0.95	(213)
77.8778	1.22665	0.56	(−1121)

**Table 4:** Characteristic transmittance infrared bands of brushite.

pH 5	Assignment [28,29,30]	pH 5	Assignment [28,29,30]
570.1	v4 of P–O–P bending	1558.48	Bending mode of H_2_O
887.11	Stretching mode of (HPO_4_ ^2−^)	1643.89	Bending mode of H_2_O
995.75	v1 stretching vibration of PO_4_ ^3−^	2391.00	Overtone or combination band
1068.31	v2 (P–O) Stretching of the PO_4_ ^3−^ group	3199.37	(O–H) stretching of water
1130.24	v′6 and v″6 of HPO_4_ ^2−^ group	3417.01	Stretching of water (v_s_ (O–H))
1402.04	Bending (O–H) in HPO_4_ ^2−^ group		

**Table 5:** Effect of different phosphorus treatments on shoot and root dry weights (g pot^−1^) of snap bean plants at 45 days after sowing during the two seasons, SI (2016–2017) and SII (2017–2018) seasons.

	Dry Weight (g pot^−1^)			
Treatments	SI		SII	
	Shoot	Root	Shoot	Root
**T1**	5.26j ± 0.11	1.49m ± 0.05	4.27k ± 0.05	1.41l ± 0.07
**T2**	6.49i ± 0.04	2.14j ± 0.03	5.49j ± 0.04	1.86jk ± 0.05
**T3**	9.14c ± 0.02	2.90d ± 0.09	7.91d ± 0.10	2.55cd ± 0.06
**T4**	7.50g ± 0.14	2.42hi ± 0.07	5.99i ± 0.19	1.94ij ± 0.07
**T5**	7.44g ± 0.09	2.03k ± 0.05	6.35g ± 0.08	1.69k ± 0.14
**T6**	7.99f ± 0.11	2.67e ± 0.08	7.04f ± 0.15	2.17gh± 0.05
**T7**	9.23c± 0.11	3.10c ± 0.11	8.40c ± 0.05	2.79b ± 0.12
**T8**	8.24e ± 0.17	2.82d ± 0.05	6.93f ± 0.08	2.28fg ± 0.07
**T9**	7.36gh ± 0.09	2.38i ± 0.08	6.16h ± 0.08	2.07hi ± 0.08
**T10**	9.72b ± 0.06	3.39b ± 0.02	8.94b ± 0.17	3.25a ± 0.10
**T11**	8.58d ± 0.22	2.65ef ± 0.04	7.64e ± 0.26	2.78b ± 0.05
**T12**	8.26e ± 0.08	2.57f ± 0.06	7.41e ± 0.09	2.38ef ± 0.07
**T13**	10.29a ± 0.12	3.51a ± 0.03	9.75a ± 0.10	3.40a ± 0.12
**T14**	8.67d ± 0.24	2.56fg ± 0.03	8.44c ± 0.09	2.47de ± 0.09
**T15**	6.46i ± 0.06	1.90l ± 0.10	5.97i ± 0.14	1.84j ± 0.12
**T16**	8.34e ± 0.10	2.51gh ± 0.07	7.85d ± 0.19	2.61c ± 0.11
**T17**	7.17h± 0.07	2.05jk ± 0.04	6.45g ± 0.23	1.92ij ± 0.18

Data are displayed as means ± standard deviation (n = 3). Different letters mean values indicate significant differences at *p* ≤ 0.05, according to the least significance difference.

**Table 6:** Effect of different phosphorus testaments on yield and its components of snap bean plants.

Treatments	No. of Pods (plant^−1^)	Pod Length (cm)	Pod Diameter (mm)	Pod Weight (g plant^−1^)	Weight of Pod (g)	Pod Yield (ton ha^−1^)
30 kg P_2_O_5_ fed^−1^ soil (T1)	14.69 ± 0.44	11.55 ± 0.12	6.50 ± 0.07	59.66 ± 1.55	3.74 ± 0.19	11.45 ± 0.30
60 Kg P_2_O_5_ fed^−1^ soil (T5)	16.27 ± 0.50	12.10 ± 0.01	7.00 ± 0.22	68.49 ± 1.97	3.98 ± 0.11	13.11 ± 0.48
T1 + 5% NCaP foliar (T3)	16.55 ± 0.88	12.46 ± 0.10	7.20 ± 0.15	73.49 ± 2.47	4.15 ± 0.21	13.78 ± 0.13
T5 + 5% NCaP foliar (T7)	17.98 ± 0.31	12.74 ± 0.12	7.25 ± 0.11	74.70 ± 0.71	4.20 ± 0.17	14.34 ± 0.12
10% NCaP soil + 5%NCaP foliar (T10)	18.47 ± 0.74	13.43 ± 0.10	7.40 ± 0.05	78.66 ± 2.29	4.31 ± 0.12	14.67 ± 0.05
20% NCaP soil + 5%NCaP foliar (T13)	20.72 ± 0.85	14.52 ± 0.38	7.60 ± 0.17	83.86 ± 4.16	4.45 ± 0.04	15.33 ± 0.01
L.S.D.	1.2950	0.3335	0.2227	4.7927	0.2960	0.3978

Data are displayed as means ± standard deviation (*n* = 3). Least Significant Difference (LSD) test at *p* ≤ 0.05.

**Table 7:** Effect of different phosphorus treatments on the nutrient concentration (N, P and K) and crude protein percentage in pods of snap bean plants.

Treatments	N%	P%	K%	Crude Protein %
30 kg P_2_O_5_ fed^−1^ soil (T1)	2.57 ± 0.08	0.35 ± 0.02	1.73 ± 0.07	16.06 ± 0.73
60 kg P_2_O_5_ fed^−1^ soil (T5)	3.01 ± 0.13	0.42 ± 0.01	2.21 ± 0.06	18.81 ± 0.81
T1+ 5% NCaP foliar (T3)	3.28 ± 0.10	0.40 ± 0.02	2.24 ± 0.07	20.50 ± 0.81
T5+ 5% NCaP foliar (T7)	3.33 ± 0.17	0.45 ± 0.02	2.33 ± 0.12	20.81 ± 1.04
10% NCaP soil + 5%NCaP foliar (T10)	3.41 ± 0.10	0.49 ± 0.01	2.65 ± 0.13	21.31 ± 1.06
20% NCaP soil + 5%NCaP foliar (T13)	3.48 ± 0.17	0.53 ± 0.02	2.91 ± 0.08	21.75 ± 0.64
LSD	0.2492	0.0319	0.1705	1.5991

Data are displayed as means ± standard deviation (*n* = 3). Least Significant Difference (LSD) test at *p* ≤ 0.05.

## Data Availability

The data presented in this study are available on request from the corresponding author.
